# Application of decision tree model in diagnosis of mycoplasma pneumoniae pneumonia with plastic bronchitis

**DOI:** 10.1186/s13052-025-01934-8

**Published:** 2025-03-24

**Authors:** Lin Li, Dong Wang, Rongrong Yang, Xing Liao, Ling Wu

**Affiliations:** Department of Infectious Diseases, College of Clinical Medicine for Obstetrics & Gynecology and Pediatrics, Fujian Children’s Hospital (Fujian Branch of Shanghai Children’s Medical Center), National Regional Medical Center, Fujian Medical University, Fuzhou, 350014 PR China

**Keywords:** Decision tree model, Mycoplasma pneumoniae, Plastic bronchitis, Childhood pneumonia, Diagnosis

## Abstract

**Background:**

To establish a decision tree model of Mycoplasma pneumoniae pneumonia(MPP) complicated with plastic bronchitis(PB) in children, and to explore the application value of decision tree model in the auxiliary diagnosis of children.

**Methods:**

A retrospective study was conducted to collect the clinical data of 214 children who met the admission criteria in Fujian Children’s Hospital from June 2022 to June 2024, and they were divided into plastic bronchitis group (*n* = 66) and non-plastic bronchitis group (*n* = 148). Using R language, 70% of the data from each group of patients was randomly selected for training the model using decision tree algorithm analysis, thus generating a clinical diagnostic decision tree for Mycoplasma pneumoniae (MP) combined with PB. The generated decision tree model was validated on the validation sample dataset and the detection effect value of the model was calculated.

**Result:**

In this study, a total of 22 indicators were employed to build the decision tree diagnostic model. Univariate statistical analysis was carried out prior to the model construction, and it was discovered that the differences of 13 indicators between the molded group and the non-molded group were statistically significant. A decision tree model with D-dimer ≥ 1.7ug/mL, C-reactive protein ≥ 15 mg/L, drug resistance or not, and serum ferritin<137 mg/L was constructed in the training sample dataset of the molded group and the non-molded group. The sensitivity of the decision tree model was 0.884, which was verified in the dataset of the remolded group and the non-molded group. The specificity was 0.727, and the area under the receiver operating characteristic curve was 0.831.

**Conclusion:**

Decision tree model can provide reference for the application of auxiliary diagnosis in children with mycoplasma pneumoniae pneumonia complicated with plastic bronchitis. The model has good discriminative ability in general, and is worthy of clinical application and further study.

## Introduction

*Mycoplasma pneumoniae* (MP) is a significant pathogen responsible for lower respiratory tract infections in children, with an infection rate of community-acquired pneumonia reaching as high as 25–30%. School-age children are particularly vulnerable to this infection [1]. Reports of refractory *Mycoplasma pneumoniae* pneumonia (RMPP) in pediatric populations have been increasing annually in recent years [2]. Some RMPP patients receiving standard glucocorticoid therapy exhibit poor responses, persistent high fever, and imaging changes that indicate disease progression, along with severe pulmonary complications both internally and externally [3]. Bronchoscopy performed on these patients has revealed the presence of plastic bronchitis (PB). The clinical hallmark of this condition is the formation of dendritic foreign bodies within the bronchial lumen, which obstructs airflow to varying degrees and can lead to partial or complete lung ventilation dysfunction, potentially resulting in life-threatening situations [4]. Early clinical diagnosis is crucial for improving prognosis; however, due to the nonspecific nature of PB’s clinical manifestations and imaging findings, early recognition remains a challenging issue faced by clinicians during T-surgery procedures [5]. Therefore, this study aims to investigate the risk factors associated with PB in children suffering from *Mycoplasma pneumoniae* pneumonia (MPP) and provide a clinical basis for early prediction and timely intervention. The decision tree model accommodates both continuous and categorical variables without necessitating complex calculations for classification. Furthermore, it elucidates the significance of key attributes influencing classification decisions; thus facilitating straightforward decision-making based on specific sample characteristics through graphical representations. Internationally, decision tree models have gained widespread application in medical research across various disease diagnoses and condition assessments [6, 7]. This study seeks to explore the utility of decision tree models in assisting diagnoses for children diagnosed with MPP complicated by PB while offering an efficient method for such diagnostic assistance.

## Methods and patients

### Study patients

Medical records of 214 children diagnosed with MPP and undergoing alveolar lavage in Fujian Children’s Hospital from June 2022 to June 2024 were collected, including demographic data, main clinical manifestations, auxiliary examination indicators, etc. According to the results of bronchoscopy, the patients were divided into plastic bronchitis group (*n* = 66) and non-plastic bronchitis group (*n* = 148).

### Inclusion criteria

(1) Meet the diagnostic criteria for MPP in the “Diagnostic and Treatment Guidelines for Child Pneumonia Caused by Mycoplasma Pneumoniae (2023 Edition)”: (1) The main clinical manifestations are fever and cough, which can be accompanied by headache, nasal discharge, sore throat, and otalgia, etc. (2) The main manifestations of X-ray or chest CT are thickening and increase of peribronchovascular textures, thickening of bronchial walls, and there may be ground-glass opacity, tree-in-bud sign, thickening of interlobular septa, reticular shadows, etc. (3) The titer of single serum MP antibody is ≥ 1:160 (PA method), and the titer of double serum MP antibody increases by 4 times or more during the course of the disease. (4) MP-DNA or RNA is positive [8]. Diagnosis can be made when the above-mentioned clinical manifestations and imaging findings are combined with one or two of (3) and (4) [8]. (2) Meet the indications for bronchoscopy in the “Chinese Pediatric Flexible Bronchoscope Technique Guidelines (2018 Edition)”: (1) Patients with chest imaging abnormalities such as atelectasis and pleural cavity lesions requiring differential diagnosis; (2) etiological diagnosis and treatment of pulmonary infectious diseases [9], and undergo fiberoptic bronchoscopy during hospitalization.

### Exclusion criteria

(1) Have a history of tuberculosis or evidence of tuberculosis infection, recurrent respiratory infection, chronic lung disease, asthma, congenital or secondary immune deficiency, liver or kidney disease, cardiovascular disease, etc.; (2) Pathogenic test results combined with infection of other pathogens. This study was approved by the Ethics Committee of our Institute (2024ETKLRK010002).

### Collect data

Clinical data of all children were collected: (1) general data, including gender, age, length of stay, duration of fever, and duration of cough. (2) clinical characteristics, including whether oxygen therapy was used, whether drug resistance was used and days of use of methylprednisolone. (3) Laboratory indicators, including White blood cell (WBC), neutrophil/lymphocyte (NE/LY), C-reactive protein (CRP), Procalcitonin (PCT), Serum ferritin (Ferr), Lactate dehydrogenase (LDH), Erythrocyte sedimentation rate (ESR), D-dimer(D-d), Creatine kinase (CK), Creatine kinase isoenzyme (CKMB), Alanine aminotransferase (ALT), Aspartate aminotransferase (AST), etc. (4) Imaging features, including whether there is atelectasis or pleural effusion.

### Establishment of decision tree model

Decision tree is a machine learning method that extracts classification rules from irregular examples and presents them in a tree structure. Each internal node represents an attribute judgment, each branch represents a judgment result output, and each leaf node ultimately represents a classification result [10]. To assess the generalization ability of the model, the data set is divided into a training set and a test set. In this research, 70% of the data is used to train the model and 30% for testing the partitioning strategy of the model. 70% of the children in the plastic and non-plastic groups are randomly selected as the training data set, and the remaining 30% are chosen as the validation data set. The establishment of the decision tree model utilizes the rpat() and rpart.lpot() functions in the rpart package of the R3.6 language, and the prune() function is employed for pruning. The training set is utilized to complete the growth of the decision tree. Considering the sample size of this study, the parent node is set at 20, the child node at 5, and the maximum growth depth at 3 layers. According to the Gini coefficient, when the sample size of the sub-subset after branching is lower than the predefined threshold, the splitting is stopped to verify the data set and evaluate the decision tree model. The critical values of optimal sensitivity and specificity are obtained through the receiver operating characteristic (ROC) curve.

### Statistical analysis

SPSS26.0 statistical analysis software was used. The Mean ± SD (Mean ± SD) was used to represent the normal distribution of quantitative data, and the median (interquart distance)[M(Q25,Q75)] was used to represent the non-normal distribution of quantitative data. The T-test or Mann-Whitney U test was used for comparison between groups. Chi-square test was used for qualitative data comparison. *P* < 0.05 was considered statistically significant.

## Results

### Comparison of general data and clinical manifestations between the two groups

In comparison to the non-PB group, the PB group exhibited a greater number of hospital days, an increased duration of fever, a higher incidence of drug-resistant MP infections, and an elevated proportion of pleural effusion, all demonstrating statistically significant differences (*p* < 0.001), as illustrated in Table 1.

**Table 1:** Comparison of general data and clinical manifestations between the two groups

Item	Classify	PB-group	Non-PB-group	χ2/Ζ	*p*
Number		66	148		
gender	male	34(35.05%)	63(64.95%)	1.475	0.225
	female	32(27.35%)	85(72.65%)		
age[M(Q_25_,Q_75_), year]		7(5 ~ 8)	6(4 ~ 8)	-1.894	0.058
Length of stay[M(Q_25_,Q_75_), day]		9(7 ~ 11)	8(7 ~ 10)	-2.568	0.01
Febrile days[M(P25,P75), day]		9(7 ~ 10)	8(6 ~ 9)	-3.45	<0.001
Cough days[M(P25,P75), day]		11(9 ~ 14.25)	11(9 ~ 13.75)	-0.782	0.434
Methylprednisolone days[M(P25,P75), day]		7(5 ~ 9)	5.5(3 ~ 7)	-3.757	<0.001
Drug resistance	yes	38(46.34%)	44(53.66%)	14.975	<0.001
	no	28(26.92%)	104(78.79%)		
Oxygen therapy	yes	11(32.35%)	23(67.65%)	0.043	0.835
	no	55(30.56%)	125(69.44%)		
Lung consolidation	yes	52(33.33%)	104(66.67%)	1.676	0.195
	no	14(24.14%)	4475.86%)		
Pleural effusion	yes	23(63.89%)	13(36.11%)	22.162	<0.001
	no	43(24.16%)	135(75.84%)		

### Comparison of laboratory indexes between the two groups

Compared with the non-PB group, the levels of C-reactive protein, calcitonin, serum ferritin, D-dimer, creatine kinase, creatine kinase isoenzyme, alanine aminotransferase and aspartate aminotransferase in PB group were higher, and the levels of lactate dehydrogenase were lower, with statistical significance (*P* < 0.01), as shown in Table 2.

**Table 2:** Comparison of laboratory indexes between the two groups

Item	PB-group	Non-PB-group	Z	*p*
Number	66	148		
WBC [M(P25,P75), 10^9/L]	7.395(5.365 ~ 9.285)	7.36(5.885 ~ 9.445)	-0.957	0.338
NE/LY [M(P25,P75)]	3.277(2.2688 ~ 4.4544)	2.3537(1.6684 ~ 3.101)	-3.339	<0.001
CRP [M(P25,P75), mg/L]	28.755(16.275 ~ 46.8325)	16.53(7.46 ~ 32.215)	-3.404	<0.001
PCT [M(P25,P75), ng/mL]	0.225(0.13 ~ 0.3925)	0.12(0.08 ~ 0.2603)	-3.778	<0.001
Ferr [M(P25,P75), mg/L]	255.75(133.775 ~ 454.05)	179.2(139.225 ~ 272.475)	-3.064	0.002
LDH [M(P25,P75), U /L]	368.5(283 ~ 491.75)	296.5(257 ~ 345.75)	-2.43	0.015
ESR [M(P25,P75), mm]	44.99(32.5 ~ 61.5)	45.9801(34 ~ 58)	-0.026	0.979
D-d [M(P25,P75), ug/mL]	1.435(0.655 ~ 3.2725)	0.735(0.495 ~ 1.1725)	-4.456	<0.001
CK [M(P25,P75), U/L]	103.5(61.75 ~ 193.5)	85.5(60 ~ 124)	-1.849	0.064
CKMB [M(P25,P75), U/L]	23(17.75 ~ 28.25)	23(19 ~ 28.75)	-0.373	0.709
ALT [M(P25,P75), U/L]	16(11.75 ~ 28.25)	13(10 ~ 18.75)	-2.852	0.004
AST [M(P25,P75), U/L]	34.5(26.5 ~ 47)	29(25 ~ 36)	-2.759	0.005

### Establishment of decision tree model

**Fig. 1:**
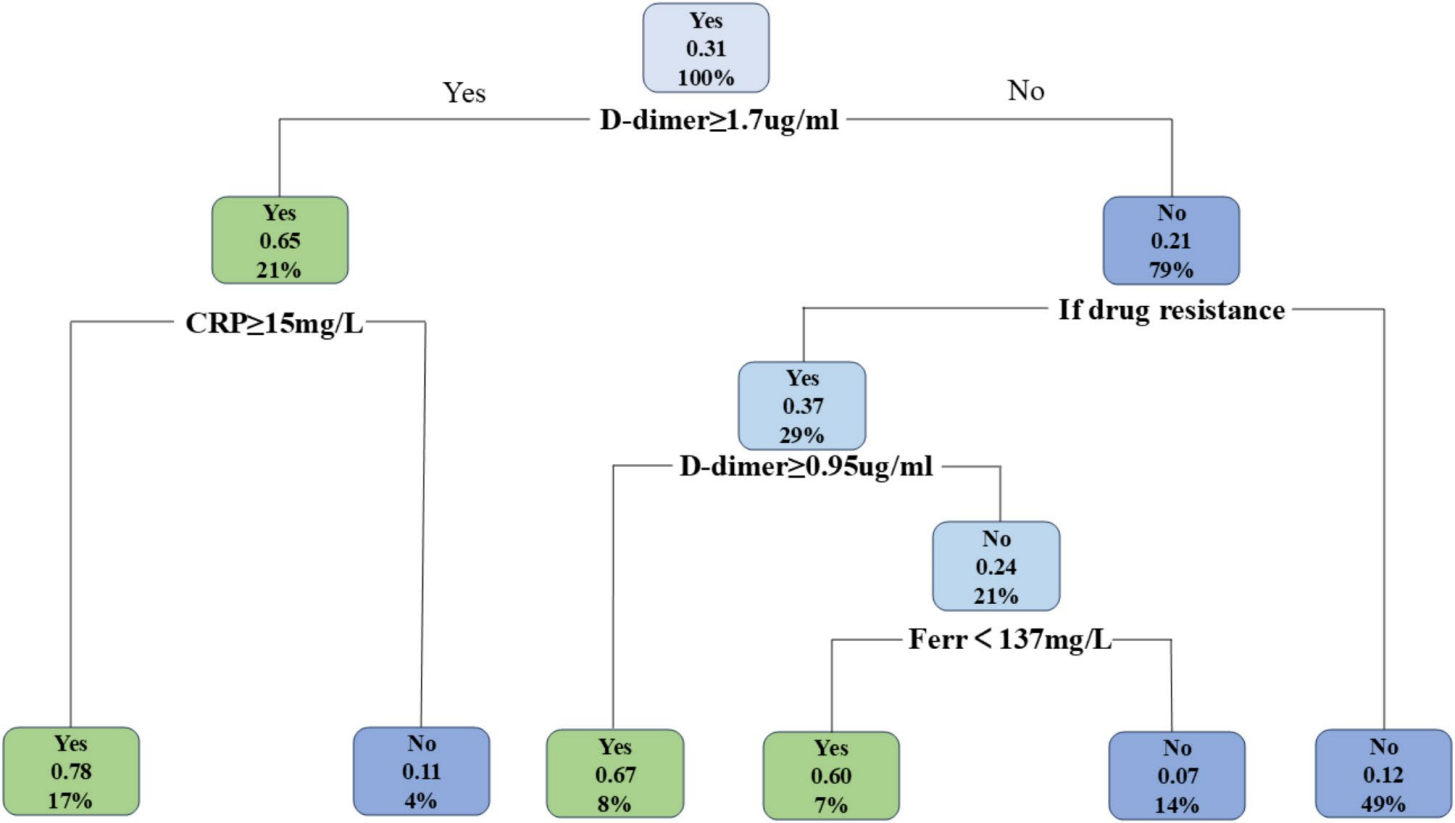
Decision tree model of mycoplasma pneumoniae pneumonia with plastic bronchitis

The 22 items of plastic and non-plastic children were imported into the rpart package in R3.6 language. The pruning strategy was used to confirm the best sensitivity and specificity. Finally, D-dimer, C-reactive protein, drug resistance and serum ferritin were used to construct the decision tree model. The model has 5 nodes and 6 endpoints. According to the decision threshold value, the two groups of children are distinguished by the judgment of whether each indicator is yes or no starting from the root node. In the training set, D-dimer was used as the feature of the first division at the root node. If D-dimer ≥ 1.7ug/mL, the prediction probability of molding was 0.65, accounting for 21% of the total sample size; for children with D-dimer ≥ 1.7ug/mL, if C-reactive protein ≥ 15 mg/L, the prediction probability of molding was 0.78. Accounting for 17% of the total sample size, if the C-reactive protein < 15 mg/L, the probability of predicting plastic shape is 0.11, accounting for 4% of the total sample size. If the D-dimer < 1.7, the probability of predicting molding is 0.21, accounting for 79% of the total sample size. For children with D-dimer < 1.7ug/mL, if there is resistance, then it is necessary to further evaluate whether the D-dimer is ≥ 0.95ug/mL. If the D-dimer is ≥ 0.95ug/mL, then the probability of predicting plastic pattern is 0.67, accounting for 8% of the total sample size. If the D-dimer is < 0.95ug/mL, the probability of predicting plastic pattern is 0.67. Therefore, it is necessary to further evaluate whether serum ferritin is < 137 mg/L. If serum ferritin is < 137 mg/L, the probability of predicting plastic pattern is 0.6, accounting for 7% of the total sample size; if serum ferritin is ≥ 137 mg/L, the probability of predicting plastic pattern is 0.07, accounting for 14% of the total sample size. For children with D-dimer < 1.7ug/mL, if no resistance is present, the probability of predicting plastic pattern is 0.12, accounting for 49% of the total sample size (Fig. 1).

**Fig. 2:**
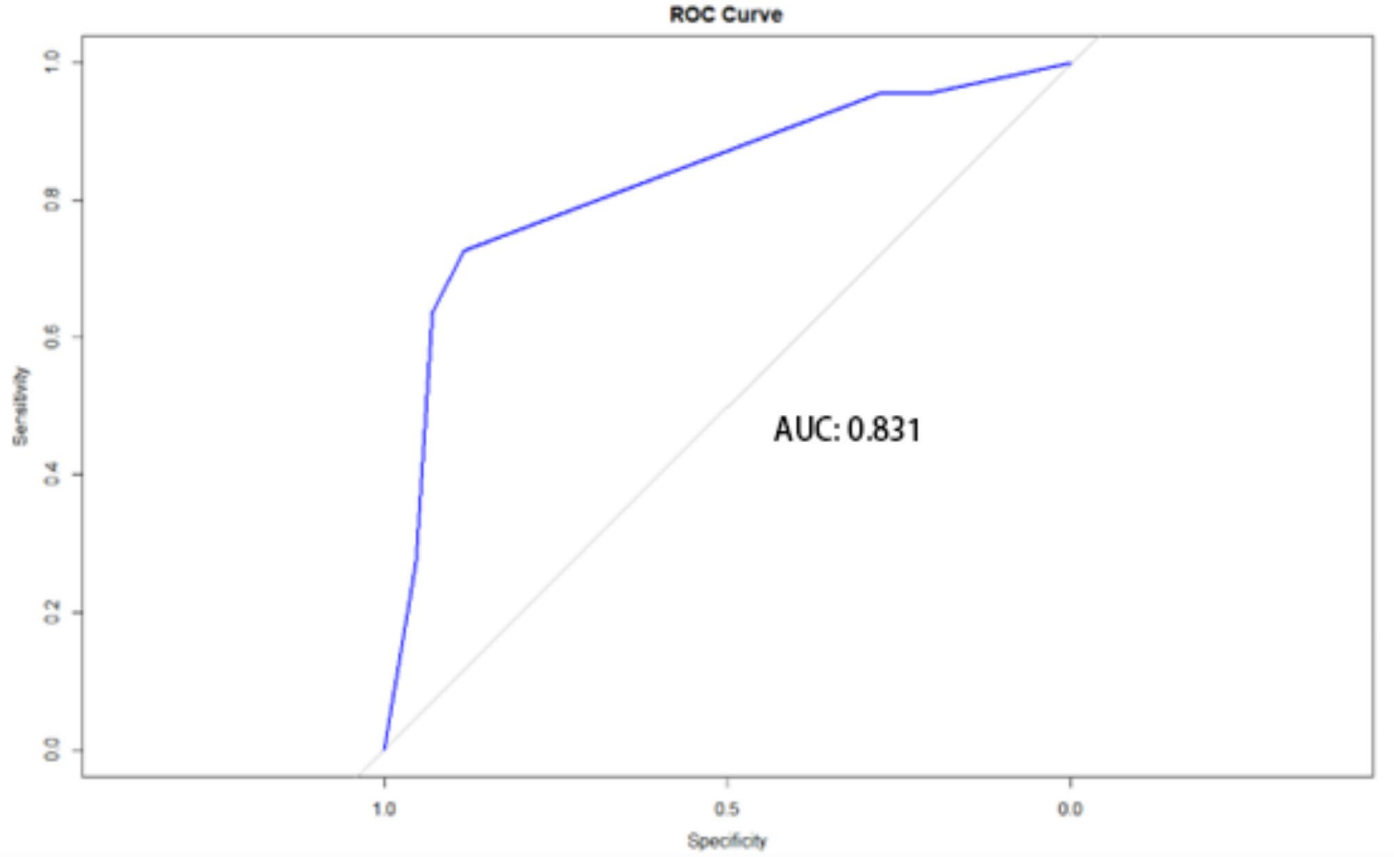
The ROC of decision tree model

In the PB and non-PB groups, 22 and 43 children (30%) were randomly selected as validation data sets, and ROC curve analysis was performed based on the above decision tree model compared with the actual discharge diagnosis of the children (Fig. 2). The area under the curve (AUC) verified by this model was 0.831. The sensitivity and specificity of the model were 0.884 and 0.727.

## Discussion

PB is a relatively rare respiratory disease that can lead to serious respiratory complications such as respiratory failure and death [11, 12]. Its etiology has not been fully elucidated, but PB is associated with several heart and lung diseases, including cyanotic congenital heart disease, asthma, cystic fibrosis, respiratory infections, sickle cell anemia, tumors, and lung transplantation [13, 14]. It has been reported that in Asia, respiratory infection is the main cause of PB, while MP is the main pathogen causing PB [15, 16]. In recent years, with the development of bronchoscopy technology, reports of plastic bronchitis have gradually increased. After the outbreak of the novel coronavirus, we also found that the incidence of MP in East China is higher than before, especially the increase of drug-resistant strains with A2063G mutation. This study found that patients infected with drug-resistant strains had a higher proportion of PB than patients infected with non-drug-resistant strains.

In this study, we found that the PB group had longer hospital stays than the non-PB group. This is consistent with the reports of Zhong et al. [5] and Hua [17], which may be due to the following two reasons. On the one hand, the clinical manifestations of PB caused by MP infection are not specific, and MPP is considered to be self-limiting. PB caused by MPP cannot be treated in time in the early stage [18]. On the other hand, the mechanism of PB after MP infection may be closely related to MP resistance, which increases the difficulty of treatment [19]. Laboratory detection index is a simple and practical method to evaluate the severity of PB. In this study, it was found that the ratio of neutrophil to lymphocyte, CRP, PCT, Ferr, D-dimer, LDH, ALT and AST in PB group were significantly increased, which was consistent with recent findings [20]. After MP infection, CRP and PCT, as sensitive indicators of the acute phase of inflammation, help to identify PB formation caused by MPP [15]. LDH is a cytoplasmic enzyme present in many vital organs and is a non-specific inflammatory biomarker of lung tissue or cell membrane injury. LDH is an important indicator for monitoring the severity of infection and inflammatory diseases [21]. Systemic inflammation caused by MP infection leads to imbalances in the clotting and anticlotting systems, resulting in a hypercoagulable state and higher D-dimer levels [22]. In addition to intrapulmonary injury, MP infection can also cause extrapulmonary injury, such as heart and liver injury [23]. CK and CKMB can reflect the degree of myocardial damage to a certain extent, while ATL and AST can reflect the degree of liver damage. The more severe the MPP children were, the higher the CK, CKMB, ALT and AST levels were. In the imaging features, we found that the incidence of pleural effusion and pericardial effusion in the PB group was higher than that in the non-PB group, which was consistent with the results of some previous studies, and the local immune response of MP patients with PB was stronger than that of non-PB patients [24, 25]. Pleural effusion is caused by severe local inflammation, lacunar obstruction, and elevated local hydrostatic pressure.

This study shows that D-d, CRP, whether the patient is resistant to treatment, and Ferr are the four important indicators used to establish a decision tree model for the diagnosis of PB in children with MP infection. Previous studies have reported that a peak temperature of > 39.0 °C in children is an independent factor for the occurrence of PB. In another study by Yang et al., lymphocyte level and pleural effusion were found to be independent risk factors for PB in children with MP and PB. Unlike previous studies, this study used a decision tree model to identify five nodes, including whether D-dimer is ≥ 1.7ug/mL, whether CRP is ≥ 15 mg/L, whether the patient is resistant to treatment, whether D-dimer is ≥ 0.95ug/ and whether serum ferritin is < 137 mg/L, which can help to quickly and accurately determine whether a child with MPP has PB. This is beneficial for early diagnosis of MPP with PB in clinical practice, allowing for more timely treatment. Of the five nodes, serum ferritin < 137 mg/L may seem less important, which may be due to the fact that in children with MPP who have not had a significant increase in D-dimer, their serum ferritin levels have not shown a significant increase either.

The main sequelae of MP-induced plastic bronchitis are BO. In some cases, early diagnosis and treatment as well as repeated bronchoscopic flushing to completely clear the airway may improve prognosis. In this study, we employed the decision tree algorithm to develop a novel diagnostic model for MPP complicated with PB. This is the first time a diagnostic model incorporating factors such as D-dimer, serum ferritin, CRP, drug resistance, and others has been established for pediatric patients with MPP combined with PB. Further validation in pediatric cases confirmed that the decision tree model exhibits high sensitivity and specificity. As a multi-factor biomarker joint evaluation system, it holds significant importance in guiding timely treatment and reducing long-term sequelae and complications of the respiratory system.

Despite these advantages, our study still has some limitations. First of all, we didn’t follow up the patients. Second, this was a single-center study; Further multicenter and large sample studies are needed to reduce bias. Third, the specific mechanism by which MPP leads to PB formation needs to be further explored. Further study will be explored above the three aspects in the future.

## Conclusion

Decision tree model can provide reference for the application of auxiliary diagnosis in children with MPP complicated with PB. The model has good discriminative ability in general, and is worthy of clinical application and further study.

## Data Availability

The datasets generated and/or analysed during the current study are not publicly available due patients’ privacy needs to be protected but are available from the corresponding author on reasonable request.

## List of abbreviations

MP: Mycoplasma pneumoniae
MPP: Mycoplasma pneumoniae pneumonia
RMPP: Reports of refractory Mycoplasma pneumoniae pneumonia
PB: plastic bronchitis
WBC: White blood cell
NE/LY: neutrophil/lymphocyte
CRP: C-reactive protein
PCT: Procalcitonin
Ferr: Serum ferritin
LDH: Lactate dehydrogenase
ESR: Erythrocyte sedimentation rate
D-d: D-dimer
CK: Creatine kinase
CKMB: Creatine kinase isoenzyme
ALT: Alanine aminotransferase
AST: Aspartate aminotransferase
ROC: receiver operating characteristic
AUC: area under the curve
